# *In vitro* effects of different levels of quebracho and chestnut tannins on rumen methane production, fermentation parameters, and microbiota

**DOI:** 10.3389/fvets.2023.1178288

**Published:** 2023-04-18

**Authors:** Marco Battelli, Stefania Colombini, Pietro Parma, Gianluca Galassi, Gianni Matteo Crovetto, Mauro Spanghero, Davide Pravettoni, Sergio Aurelio Zanzani, Maria Teresa Manfredi, Luca Rapetti

**Affiliations:** ^1^Department of Agricultural and Environmental Sciences - Production, Landscape, Agroenergy, University of Milan, Milan, Italy; ^2^Department of Agricultural, Food, Environmental and Animal Sciences, University of Udine, Udine, Italy; ^3^Department of Veterinary Medicine and Animal Sciences, University of Milan, Lodi, Italy

**Keywords:** condensed tannins, hydrolysable tannins, methane, microbiota, digestibility, ruminants

## Abstract

Both condensed and hydrolysable tannins (CTs and HTs, respectively) have the ability to reduce enteric CH_4_ production in ruminants. However, the precise mechanism of action is not fully understood. Among the proposed hypotheses are the reduction of ruminal digestibility, direct control action on protozoa, reduction of archaea, and a hydrogen sink mechanism. In this *in vitro* study, which simulated rumen fermentation, two additives, one containing CTs (70% based on DM) from quebracho and one with HTs (75% based on DM) from chestnut, at four levels of inclusion (2, 4, 6, 8% on an as-fed basis) were added to the fermentation substrate and tested against a negative control. Both types of tannins significantly reduced total gas (GP) and CH_4_ (ml/g DM) production during the 48 h of incubation. The lower GP and CH_4_ production levels were linked to the reduction in dry matter digestibility caused by CTs and HTs. Conversely, no significant differences were observed for the protozoan and archaeal populations, suggesting a low direct effect of tannins on these rumen microorganisms *in vitro*. However, both types of tannins had negative correlations for the families Bacteroidales_BS11 and F082 and positive correlations for the genera *Prevotella* and *Succinivibrio*. Regarding the fermentation parameters, no differences were observed for pH and total volatile fatty acid production, while both CTs and HTs linearly reduced the NH_3_ content. CTs from quebracho were more effective in reducing CH_4_ production than HTs from chestnut. However, for both types of tannins, the reduction in CH_4_ production was always associated with a lower digestibility without any changes in archaea or protozoa. Due to the high variability of tannins, further studies investigating the chemical structure of the compounds and their mechanisms of action are needed to understand the different results reported in the literature.

## 1. Introduction

The livestock sector contributes to approximately 14.5% of anthropogenic greenhouse gas (GHG) emissions, and one of the most important GHGs is methane (CH_4_) (1). Ruminants contribute significantly to CH_4_ emissions due to enteric fermentation, accounting for 17% of global CH_4_ emissions (2). Moreover, ruminants release nitrogen into the environment in the form of ammonia (NH_3_) and nitrous oxide (N_2_O), another powerful GHG. Both CH_4_ and nitrogen emissions are not only related to environmental problems but are also associated with energy and organic matter losses that hamper the efficiency and productivity of farms (3).

Among the different strategies to reduce the environmental impact of ruminants, tannins have been evaluated for their potential in reducing enteric methanogenesis (4). Tannins are polyphenolic substances with great chemical variability, and in terrestrial plants, they are classified into two groups: condensed tannins (CTs) and hydrolysable tannins (HTs). Condensed tannins are polymers of flavonoids with high molecular weights, while HTs are polyesters of gallic acid and various individual sugars and are characterized by lower molecular weights than CT (5). Condensed tannins are mostly found in the vacuole and in the epidermal or subepidermal layers of leaves and fruit, while HTs accumulate in the cell wall. However, tannins are also frequently found in other plant tissues, such as bark, wood, roots, seeds and fruits. Furthermore, tannin concentrations vary according to the plant species and genotype, tissue developmental stage, and environmental conditions (6).

As reported by Mueller-Harvey et al. (7), despite the widespread occurrence of tannins (both CTs and HTs) in the plant kingdom, there are still large gaps in our knowledge regarding their role in plants and their impact on animals. In the diets of ruminants, it is possible to include tannins due to the use of naturally tannin-rich forages, such as some legumes, including sulla (*Hedysarum coronarium*), birdsfoot trefoil (*Lotus corniculatus*) and sainfoin (*Onobrychis viciifolia*), or due to the addition of purified tannin extract. Some of the most popular purified tannin extracts come from mimosa (*Mimosa tenuiflora*) and quebracho (*Schinopsis balansae*) for CTs, while a common HTs source is chestnut (*Castanea* sp.) (8).

Depending mostly on their concentrations in the diet but also on animal species and other factors (such as the chemical structure of the tannins, dietary composition and interaction with other compounds, adaptation of the ruminant to the presence of tannins, etc.), tannins could have both adverse and positive effects on ruminants (9).

One of the positive effects of tannins is the reduction of CH_4_ emitted from ruminal fermentation (3, 10, 11). This effect has been observed in several *in vitro* and *in vivo* trials, but there are some aspects that need to be explored, such as the effect of different dietary concentrations and the effect of different sources and types of tannins. In this regard, Jayanegara et al. (8) found a greater and more constant effect of HTs in comparison with CTs in reducing CH_4_ emissions with less adverse effects on digestibility. As highlighted by several authors (4, 5, 12, 13), it is necessary to investigate whether the reduction in CH_4_ emissions is linked to a reduction in the digestibility of organic matter, particularly fiber. Moreover, tannins can exert a control action on both archaea and protozoa, which are related to methane production in the rumen (14–18).

Due to their high affinity for proteins and the formation of tannin-protein complexes, tannins can also cause a reduction in protein degradability in the rumen (19). The consumption of CTs and HTs by ruminants can reduce the production of ruminal ammonia, lowering urinary nitrogen excretion and increasing faecal nitrogen excretion (7, 20, 21). This shift can improve the soil nitrogen status and reduce N_2_O emissions and nitrogen leachate into groundwater.

The negative effects include reduced palatability of the diet associated with the reduction of dry matter intake but also the reduction of feed digestibility. Additionally, when tannins are fed in high concentrations, they can be toxic to the animal (22).

A common generalization is that with tannin concentrations lower than 5% of the feed dry matter, there are mainly positive effects on ruminants, while with higher concentrations, negative effects prevail. However, it is important to consider the type of tannin, the plant from which it derives, as well as the type of diet and the species of ruminant (e.g., goats are more adapted to tannins compared to cows) (23). Furthermore, high concentrations of tannins in the diet would be difficult to apply commercially due to the high cost of tannin extracts.

The study aims to determine the effects of the dietary inclusion level of purified CTs and HTs on total gas and CH_4_ production, ruminal fermentation parameters and rumen microbial population by an *in vitro* method. The use of several regular dose levels for both types of tannins in this paper aims to allow a regression study that could help predict the effects of tannins at any low practical dietary dosage.

## 2. Materials and methods

### 2.1. Experimental design: samples and tannins tested

In this study, we evaluated the effects of the inclusion of commercial purified extracts of CTs (Silvafeed® Q powder, Silvateam, Mondovì, Italy; minimum tannin content of 70% based on DM, ISO 14088) from quebracho (*Schinopsis lorentzii* Engl.) and HTs (Silvafeed® ENC powder, Silvateam, Mondovì, Italy; minimum tannin content of 75% based on DM, ISO 14088) from chestnut (*Castanea sativa* Mill.) *in vitro*. Both types of tannin extracts were added to the fermentation substrate at different levels of inclusion: 2, 4, 6, and 8% on an as-fed basis. The fermentation substrate was composed (g/kg) of second-cut meadow hay (447), pelleted alfalfa hay (147), pea protein seeds (147), commercial concentrate mix (17% CP on DM, 220), and a vitamin–mineral mix (9) and contained 159, 391, 17.4 and 76.9 g/kg DM of CP, aNDFom, EE, and ash, respectively.

All treatments were tested versus a negative control (substrate without CT or HT addition). All treatments were tested in triplicate in two incubation runs.

### 2.2. *In vitro* gas production technique

Animal procedures were conducted under the approval of the University of Milan Ethics Committee for animal use and care and with the authorization of the Italian Ministry of Health, authorization no. 904/2016-PR.

Ruminal fluid was collected from two fistulated dry Italian Friesian dairy cows fed a diet composed of 66% hay and 34% concentrate. Depending on the treatments, 300 mg of fermentation substrate and the relative quantity of tannin inclusion (0–2–4–6–8%) were weighed into 120 ml serum bottles.

The fermentation medium was prepared according to Menke and Steingass (24) following the procedures previously described by Pirondini et al. (25). After the addition of 40 ml of the fermentation medium, the headspace of the incubation was flushed with N_2,_ and then the incubation vials were hermetically closed with rubber tops and placed in a shaking water bath (80 RPM) at 39.5°C for 48 h.

Gas production (GP) was determined by measuring the headspace pressure in the incubation vials (26). The pressure was recorded after 24 and 48 h of incubation using a digital manometer (model 840,082, Sper Scientific, Scottsdale, AZ, United States). After the headspace pressure was measured, at each reading time, 5 ml of gas was collected from each vial using a gastight syringe and stored in airtight vials for subsequent methane analysis. At the 24-h incubation interval, after gas collection, the pressure inside each vial was brought back to the atmospheric value using needles to avoid high headspace pressures that could compromise normal microbial activity (26).

### 2.3. Gas production calculation and methane measurement

The gas pressure data recorded at 24 and 48 h were converted into ml of gas produced using the ideal gas law. The values of each treatment were subsequently corrected for their respective blanks.

The methane concentration at 24 and 48 h of incubation was determined by injecting 250 μl of gas into a Varian CP-3800 gas chromatograph (Varian Chromatography Systems, Walnut Creek, CA, United States) equipped with a Supelco (2.3 m length x 2.1 mm internal diameter) stainless steel column packed with 60/80 mesh Carboxen™-1,000 stationary phase (Supelco, Bellefonte, PA, United States), using He as the carrier. The CH_4_ volume (ml) produced during the first (0–24 h) and second (24–48 h) phases of fermentation and the final cumulative volume were calculated as reported by Tavendale and colleagues (16).

### 2.4. Ruminal fermentation parameters, protozoal count, and microbiota analysis

At the end of the incubation, all vials were put into an ice bath to stop the fermentation, and the pH was recorded. Two of the three replicates per treatment were intended for the determination of VFA production, NH_3_ concentration and dry matter digestibility (DMD), while the remaining replicate was intended for the count of protozoa and the study of the microbiota.

Two of the three replicates per treatment were individually transferred into 50 ml Falcon tubes and centrifuged at 10,000 × *g* for 15 min at 4°C. After this first centrifugation, 5 and 30 ml of supernatant were sampled for subsequent VFA and NH_3_ analyses, respectively. The incubation vials were carefully washed with distilled water to remove all substrate residuals, and the contents were transferred into the respective Falcon tubes for a second centrifugation at 10,000 × *g* for 5 min at 4°C. After the second centrifugation, the supernatants were discharged, and the Falcon tubes with the precipitated residue were placed in an oven at 60°C until constant weight for the determination of the undigested DM.

For VFA determination, 1 ml of 25% meta-phosphoric acid was added to 5 ml of supernatant as described by Colombini et al. (27). After 30 min, the mixture was centrifuged again at 3,500 × *g* for 10 min. VFA analysis was performed by injecting 2 μl of the supernatant into a Varian CP-3800 gas chromatograph (Varian Chromatography Systems, Walnut Creek, CA, United States) using a Nukol fused silica capillary column (30 m length; 0.25 mm diameter; 0.25 μm film thickness; Supelco) as reported by Pirondini et al. (28).

The NH_3_ concentration was determined with the Kjeldahl method, with only the distillation and titration, using a Raypa NP-1500-MP Kjeldahl distiller (Raypa, Terrassa, Spain).

At the end of the incubation, the third replicate was used for the protozoal count and the microbiota analysis. Each vial was shaken to obtain a homogeneous solution, and then, for the protozoal count, 5 ml of fermentation medium was sampled, added to 5 ml of formalin and stored at room temperature, while for the microbiota analysis, 10 ml of fermentation medium was sampled and stored at −80°C pending extraction.

The protozoal count was performed as described by Dehority (29).

The DNA from the rumen fluid was extracted using the NucleoSpin Soil kit (Macherey-Nagel, Germany) following the procedures and using the reagents suggested by the kit manufacturer. Amplification of the V4 region of the 16S gene was conducted as described by Parada et al. (30). DNA was diluted at 10 ng/μl. For amplification, the following primers were used: 515F-Y: 5′-GTGYCAGCMGCCGCGGTAA and 926R: 5′-CCGYCAATTYMTTTRAGTTT. The amplifications were performed using 5 μl of the extracted DNA in a final reaction volume of 25 μl using Platinum Taq DNA polymerase high fidelity (Thermo-Fisher, MA, United States) following the manufacturer’s instructions. The amplifications were performed for 27 cycles using 55°C as the annealing temperature. The libraries were purified with Beads Amplure XP 0.8X and amplified with Indexes Nextera XT Illumina; they were normalized, mixed, and loaded on the MiSeq platform using the 2 × 300 bp (paired-end) approach to generate a minimum of 50,000 sequences (±20%). The raw sequences R1 and R2 (raw reads) were verified and filtered by quality, trimmed by the primers, and fused by QIIME2 v8 software. DADA2 (Qiime2) software isolated the amplicon sequence variants (formerly operational taxonomic units), whose sequences were compared against the SILVA Database SSU r123 to obtain the taxonomic assignment.

### 2.5. Statistical analysis

The data were statistically analyzed by the MIXED procedure of SAS 9.4 (SAS Institute Inc., Cary, NC, United States), with the run as a random effect, using the following factorial model:


$$Y_{ijk}=\mu +T_{i}+L_{j}+T\times L+R_{k}+\varepsilon_{ijk}$$

where *Y_ijk_* is the dependent variable, *μ* is the overall mean, *T_i_* is the tannin type effect (*i* = CTs from quebracho or HTs from chestnut), *L_j_* is the level effect (*j* = 0, 2, 4, 6, 8%), *T*×*L* is the interaction between the fixed effects, *R_k_* is the random effect of the run (*k* = 1, 2), and *e_ijk_* is the residual error. Least squares mean estimates are reported. Differences between least square means were evaluated using Tukey’s method for comparison when the *p* value of the interaction *T*×*L* was <0.10.

The linear effect was tested separately for each type of tannin using the MIXED procedure of SAS 9.4 as described by St-Pierre (31), applying the following model:


$$Y_{ij}=a+bL_{i}+R_{j}+\varepsilon_{ij}$$

where *a* represents the overall intercept, *b* is the overall regression coefficient, *L_i_* is the level of tannin inclusion (*i* = 0, 2, 4, 6, 8), *R_j_* is the random effect of the run (*j* = 1,2), and *e_ij_* is the residual error. The coefficient of determination (R^2^) and root mean square error (RMSE) were derived from single factor regression analysis between the adjusted values derived from the mixed model and the measured values (31, 32).

The quadratic and cubic effects of tannin inclusion were also tested separately for each type of tannin; however, these effects, which were not always significant, are not reported in the tables.

## 3. Results

### 3.1. Gas and methane production

The values of total gas and CH_4_ produced and the DMD are reported in Table 1, while in Tables 2, 3, the relationship between the level of tannin inclusion and the total gas and CH_4_ productions and DMD are reported for each type of tannin, CTs and HTs.

**Table 1:** Gas production, methane production and diet digestibility.

	Condensed tannin (CT) extract					Hydrolysable tannin (HT) extract					SE	*p* value		
% Tannin extract	0	2	4	6	8	0	2	4	6	8		*T* ^1^	*L* ^2^	*T×L* ^3^
GP^4^ 0–24 h (ml/g DM^5^)	161	158	153	148	147	161	156	154	153	147	1.49	0.454	<0.001	0.257
GP 24–48 h (ml/g DM)	48.1^abc^	46.8^abcd^	45.6^bcd^	43.0^d^	44.1^cd^	48.1^abc^	49.6^ab^	50.2^a^	49.9^a^	50.1^a^	0.726	<0.001	0.165	0.007
GP 0–48 h (ml/g DM)	209^a^	205^ab^	199^abc^	191^c^	191^c^	209^a^	206^ab^	205^ab^	203^ab^	197^bc^	1.90	0.003	<0.001	0.066
CH_4_ 0–24 h (ml/g DM)	46.4	44.7	42.2	39.9	38.4	46.4	43.4	43.0	41.2	40.0	0.887	0.428	<0.001	0.519
CH_4_ 24–48 h (ml/g DM)	6.85	7.11	6.53	6.59	5.81	6.85	7.89	8.17	8.38	7.15	0.504	0.007	0.254	0.432
CH_4_ 0–48 h (ml/g DM)	53.2	51.8	48.8	46.5	44.2	53.2	51.3	51.1	49.6	47.2	1.16	0.061	<0.001	0.417
CH_4_ 0–24 h (% GP)	28.8	28.3	27.6	27.0	26.2	28.8	27.9	27.9	27.0	27.3	0.459	0.531	0.010	0.573
CH_4_ 24–48 h (% GP)	14.2	15.2	14.3	15.3	13.1	14.2	16.0	16.3	16.8	14.3	0.958	0.110	0.168	0.873
CH_4_ 0–48 h (% GP)	25.4	25.3	24.5	24.4	23.2	25.4	25.0	25.0	24.5	24.0	0.454	0.464	0.021	0.752
DMD^6^ (%)	71.3^a^	70.3^ab^	68.5^bc^	68.2^bc^	67.4^cd^	71.3^a^	71.0^a^	69.7^abc^	68.1^bc^	65.1^d^	0.420	0.800	<0.001	0.022
GP 0–48 h (ml/g dDM^7^)	294	292	290	285	284	294	289	293	298	303	4.62	0.052	0.955	0.194
CH_4_ 0–48 h (ml/g dDM)	277	275	263	253	244	277	267	270	268	267	7.22	0.129	0.093	0.300

^a,b,c,d^Different letters on the same row correspond to different least squares means after Tukey’s adjustment (*p* < 0.05).

1 *T*: *p* value of the type of tannin effect, CT vs. HT.

2 *L*: *p* value of the level of inclusion effect.

3 *T*×*L*: *p* value of the interaction of the fixed effects.

4 GP: gas production.

5 DM: dry matter.

6 DMD: dry matter digestibility.

7 dDM: digested dry matter.

**Table 2:** Prediction equations of the gas production based on the inclusion level of the condensed tannin.*

	*a*	SE	*p* value	*b*	SE	*p* value	RMSE	R^2^
GP^1^ 0–24 h (ml/g DM^2^)	161	5.41	0.021	−1.93	0.233	<0.001	0.987	0.968
GP 24–48 h (ml/g DM)	47.9	1.19	0.016	−0.590	0.128	0.003	0.741	0.835
GP 0–48 h (ml/g DM)	209	6.55	0.020	−2.52	0.349	<0.001	1.675	0.947
CH_4_ 0–24 h (ml/g DM)	46.5	0.693	0.010	−1.04	0.142	<0.001	0.239	0.994
CH_4_ 24–48 h (ml/g DM)	7.10	0.265	0.024	−0.131	0.050	0.035	0.234	0.712
CH_4_ 0–48 h (ml/g DM)	53.6	0.867	0.010	−1.17	0.169	<0.001	0.308	0.991
CH_4_ 0–24 h (% GP)	28.9	0.787	0.017	−0.326	0.067	0.002	0.068	0.995
CH_4_ 24–48 h (% GP)	14.9	0.532	0.023	−0.103	0.109	0.379	0.742	0.138
CH_4_ 0–48 h (% GP)	25.7	0.599	0.015	−0.274	0.065	0.004	0.256	0.897
DMD^3^ (%)	71.1	0.368	0.003	−0.498	0.058	<0.001	4.875	0.949
GP 0–48 h (ml/g dDM^4^)	294	9.77	0.021	−1.32	0.594	0.061	0.819	0.959
CH_4_ 0–48 h (ml/g dDM)	280	5.24	0.012	−4.43	1.07	0.004	2.235	0.969

In the model (*Y* = *a* + *bX*), *a* and *b* represent the intercept and the regression variable coefficient, respectively.

* Predictions of gas production, methane production and diet digestibility based on the level of the condensed tannin extract inclusion as an independent regression variable (*X*) according to a linear mixed effect regression model with the run as a random effect.

1 GP: gas production.

2 DM: dry matter.

3 DMD: dry matter digestibility.

4 dDM: digested dry matter.

**Table 3:** Prediction equations of the gas production based on the inclusion level of the hydrolysable tannin.*

	*a*	SE	*p* value	*b*	SE	*p* value	RMSE	R^2^
GP^1^ 0–24 h (ml/g DM^2^)	161	6.28	0.025	−1.58	0.232	<0.001	1.16	0.935
GP 24–48 h (ml/g DM)	48.7	1.96	0.026	0.213	0.086	0.042	0.469	0.627
GP 0–48 h (ml/g DM)	209	8.18	0.025	−1.37	0.213	<0.001	1.211	0.911
CH_4_ 0–24 h (ml/g DM)	45.8	0.911	0.013	−0.750	0.092	<0.001	0.502	0.947
CH_4_ 24–48 h (ml/g DM)	7.47	0.512	0.044	0.055	0.105	0.617	0.570	0.068
CH_4_ 0–48 h (ml/g DM)	53.3	1.08	0.013	−0.695	0.142	0.002	0.495	0.939
CH_4_ 0–24 h (% GP)	28.5	0.630	0.014	−0.193	0.059	0.013	0.279	0.797
CH_4_ 24–48 h (% GP)	15.3	0.989	0.041	0.056	0.202	0.790	1.059	0.018
CH_4_ 0–48 h (% GP)	25.5	0.600	0.015	−0.168	0.062	0.030	0.151	0.905
DMD^3^ (%)	72.1	0.536	0.005	−0.762	0.109	<0.001	0.691	0.908
GP 0–48 h (ml/g dDM^4^)	290	13.2	0.029	1.32	0.595	0.062	2.841	0.644
CH_4_ 0–48 h (ml/g dDM)	274	5.65	0.013	−0.952	0.791	0.268	2.635	0.510

In the model (*Y* = *a* + *bX*), *a* and *b* represent the intercept and the regression variable coefficient, respectively.

* Predictions of gas production, methane production and diet digestibility based on the level of hydrolysable tannin extract inclusion as an independent regression variable (*X*) according to a linear mixed effect regression model with the run as a random effect.

1 GP: gas production.

2 DM: dry matter.

3 DMD: dry matter digestibility.

4 dDM: digested dry matter.

The GP from 0 to 24 h of incubation was significantly reduced by the level of inclusion of the tannins (*p* < 0.001), showing a linear reduction of the GP (Tables 2, 3) as the level of inclusion for both types of tannins increased.

During the time interval between 24 and 48 h of incubation, CTs continued to reduce the GP, while HTs had the opposite trend, numerically increasing the GP compared to the control, as shown by the different signs of the slope values in Tables 2, 3 (−0.590 and 0.213 for CTs and HTs, respectively; *p* = 0.003 and *p* = 0.042 for CTs and HTs, respectively).

Considering the total GP during the 48 h of incubation and comparing the two treatments at the different levels of inclusion, CTs had a greater effect in reducing GP than HTs (*p* = 0.066). This was confirmed by the slope values of the two equations of prediction (−2.52 and − 1.37 for CTs and HTs, respectively, for both types of tannins *p* < 0.001, Table 2).

The CH_4_ production (ml/g DM) at 0–24 and 0–48 h of incubation was reduced as the level of inclusion increased for both types of tannins (*p* < 0.001). However, during the second day of incubation (24–48 h), only CTs linearly reduced CH_4_ production (slope value of −0.131, *p* = 0.035, Table 2).

The percentage of CH_4_ in total GP was significantly lowered by the level of inclusion of both types of tannins at both 0–24 h (*p* = 0.010) and 0–48 h of incubation (*p* = 0.021; Table 1). The methanogenesis reduction effect was more pronounced with CTs than with HTs, as can be observed by the slope values of the two tannins for the percentage of CH_4_ against total GP (0–48 h; −0.274 for CTs, *p* = 0.004 and − 0.168 for HTs, *p* = 0.030).

The DMD was significantly reduced as the level of tannin inclusion increased for both CTs and HTs (*p* < 0.001). From the two regression equations (Tables 2, 3), it is possible to calculate that the addition of one percentage point of tannins caused a DMD reduction of 0.498% (for CTs) and 0.762% (for HTs).

### 3.2. Ruminal fermentation parameters

The ruminal fermentation parameters are reported in Table 4, while in Tables 5, 6, the relationships (expressed as a linear regression) between the level of tannin inclusion and the fermentation parameters are reported for each type of tannin, CTs and HTs.

**Table 4:** Ruminal fermentation parameters.

	Condensed tannin (CT) extract					Hydrolysable tannin (HT) extract					SE	*p* value		
% Tannin extract	0	2	4	6	8	0	2	4	6	8		*T* ^1^	*L* ^2^	*T×L* ^3^
pH	6.85	6.85	6.86	6.86	6.86	6.85	6.84	6.84	6.84	6.83	0.006	<0.001	0.496	0.187
NH_3_ (mg/dl)	34.2	31.5	30.4	29.3	28.4	34.2	32.8	31.0	29.6	28.1	1.03	0.568	0.002	0.942
Total VFA^4^ (mmol/L)	81.3	78.4	76.0	75.5	73.2	81.3	78.6	76.2	73.5	69.3	3.64	0.638	0.152	0.969
VFA, mol/100 mol VFA														
Acetate	62.4	61.3	61.5	62.4	62.7	62.4	61.4	62.3	62.7	63.8	0.728	0.364	0.191	0.935
Propionate	17.7	18.2	18.9	18.1	18.6	17.7	18.5	17.6	17.8	17.8	0.532	0.237	0.761	0.617
Isobutyrate	1.44	1.49	1.54	1.32	1.39	1.44	1.39	1.38	1.33	1.26	0.057	0.068	0.095	0.489
n-butyrate	13.9	14.4	13.5	13.8	12.9	13.9	14.2	14.4	14.2	13.5	0.448	0.279	0.202	0.738
Butyrate	15.4	15.9	15.1	15.2	14.3	15.4	15.6	15.8	15.5	14.7	0.435	0.385	0.130	0.768
Isovalerate	2.75	2.81	2.57	2.69	2.83	2.75	2.50	2.53	2.36	2.14	0.171	0.032	0.567	0.334
n-valerate	1.79	1.76	1.89	1.58	1.65	1.79	1.97	1.74	1.65	1.56	0.130	0.920	0.236	0.672
Valerate	4.54	4.57	4.46	4.27	4.49	4.54	4.48	4.27	4.01	3.70	0.220	0.089	0.214	0.470
Acetate:propionate	3.53	3.37	3.25	3.44	3.39	3.53	3.33	3.53	3.52	3.59	0.127	0.220	0.613	0.706

^a,b^Different letters on the same row correspond to different least squares means (p < 0.05).

1 *T*: *p* value of the type of tannin effect, CT vs. HT.

2 *L*: *p* value of the level of inclusion effect.

3 *T*×*L*: *p* value of the interaction of the fixed effects.

4 VFA: volatile fatty acids.

**Table 5:** Prediction equations of the fermentation parameters based on the inclusion level of the condensed tannin.*

	*a*	SE	*p* value	*b*	SE	*p* value	RMSE	R^2^
pH	6.85	0.006	<0.001	0.001	0.001	0.222	0.003	0.750
NH_3_ (mg/dl)	33.5	0.576	0.011	−0.697	0.118	<0.001	0.484	0.942
Total VFA^1^ (mmol/L)	80.7	4.32	0.034	−0.946	0.213	0.003	0.564	0.958
VFA, mol/100 mol VFA								
Acetate	61.7	0.632	0.007	0.090	0.098	0.388	0.499	0.189
Propionate	18.0	0.496	0.018	0.078	0.073	0.319	0.338	0.336
Isobutyrate	1.49	0.097	0.041	−0.013	0.013	0.334	0.066	0.249
n-butyrate	14.3	0.450	0.020	−0.135	0.085	0.155	0.336	0.554
Butyrate	15.8	0.499	0.020	−0.149	0.081	0.110	0.319	0.624
Isovalerate	2.72	0.405	0.094	0.003	0.028	0.926	0.094	0.004
n-valerate	1.83	0.153	0.053	−0.023	0.021	0.315	0.087	0.359
Valerate	4.54	0.533	0.074	−0.020	0.033	0.562	0.089	0.289
Acetate:propionate	3.44	0.081	0.015	−0.010	0.016	0.567	0.087	0.105

In the model (*Y* = *a* + *bX*), *a* and *b* represent the intercept and the regression variable coefficient, respectively.

* Predictions of fermentation parameters based on the level of condensed tannin extract inclusion as an independent regression variable (*X*) according to a linear mixed effect regression model with the run as a random effect.

1 VFA: volatile fatty acids.

**Table 6:** Prediction equations of the fermentation parameters based on the inclusion level of the hydrolysable tannin.*

	*a*	SE	*p* value	*b*	SE	*p* value	RMSE	R^2^
pH	6.85	0.011	0.001	−0.002	0.0011	0.0561	0.003	0.800
NH_3_ (mg/dl)	34.3	0.687	0.013	−0.778	0.140	<0.001	0.088	0.998
Total VFA^1^ (mmol/L)	81.6	9.42	0.073	−1.45	0.405	0.009	0.471	0.987
VFA, mol/100 mol VFA								
Acetate	61.7	0.915	0.009	0.202	0.126	0.151	0.511	0.563
Propionate	18.0	0.327	0.012	−0.030	0.0668	0.672	0.311	0.049
Isobutyrate	1.44	0.116	0.051	−0.021	0.004	0.001	0.082	0.948
n-butyrate	14.2	0.280	0.013	−0.046	0.055	0.438	0.486	0.130
Butyrate	15.7	0.368	0.015	−0.066	0.057	0.289	0.309	0.321
Isovalerate	2.729	0.211	0.049	−0.068	0.009	<0.001	0.347	0.911
n-valerate	1.90	0.144	0.048	−0.039	0.019	0.076	0.095	0.635
Valerate	4.63	0.339	0.047	−0.107	0.019	<0.001	0.067	0.953
Acetate:propionate	3.44	0.102	0.019	0.016	0.019	0.434	0.145	0.245

In the model (*Y* = *a* + *bX*), *a* and *b* represent the intercept and the regression variable coefficient, respectively.

* Predictions of fermentation parameters based on the level of hydrolysable tannin extract inclusion as an independent regression variable (*X*) according to a linear mixed effect regression model with the run as a random effect.

1 VFA: volatile fatty acids.

Regarding the pH, a significant effect of the treatment (6.86 vs. 6.84 for CTs and HTs, respectively; *p* < 0.001) was recorded; however, the difference was very little.

The NH_3_ concentration was significantly reduced (*p* = 0.002) with increasing tannin level for both types of tannins, as also shown by the slope coefficients (−0.697 and −0.778 for CTs and HTs, *p* < 0.001 for both types of tannins).

When all treatments were studied together, no differences were recorded for the total VFA production between the type of tannin and the level of inclusion. However, considering CTs and HTs separately, a linear reduction of the total VFA production for both types of tannins was observed (*b* coefficient − 0.946, *p* = 0.003, R^2^ = 0.958; for CTs; *b* coefficient − 1.45, *p* = 0.009, R^2^ = 0.987 for HTs).

No differences among treatments were observed for the contents of acetate, propionate, and butyrate expressed as a percentage of total VFA. However, isovalerate was significantly affected by the type of tannin (2.73 vs. 2.43 for CTs and HTs, *p* = 0.032), and a treatment trend was found for isobutyrate and valerate: HTs, unlike CTs, linearly reduced isobutyrate, isovalerate, n-valerate, and valerate as the level of inclusion increased, as can be observed from the slope coefficients in Tables 5, 6.

### 3.3. Protozoal count and microbiota analysis

In Table 7, the number of total protozoa and the percentages of *Entodinium* and *Holotricha* are reported. No level of inclusion, for both CTs and HTs, significantly influenced the number of protozoa compared to the control; however, CTs significantly increased the number of total protozoa compared to HTs (383 vs. 331 × 10^3^ cell/ml, *p* = 0.034, for CTs and HTs, respectively). The inclusion of tannins did not affect the percentage composition of protozoa.

**Table 7:** Protozoa count and relative abundance of Entodinium and Holotrica.

	Condensed tannin (CT) extract					Hydrolysable tannin (HT) extract					SE	*p* value		
% Tannin extract	0	2	4	6	8	0	2	4	6	8		*T* ^1^	*L* ^2^	*T*×*L*^3^
Total protozoa (×10^3^ cell/ml)	318	397	398	390	414	318	380	317	308	332	33.2	0.034	0.340	0.582
Entodinium (% total protozoa)	98.1	97.3	98.9	98.8	98.8	98.1	98.7	98.2	97.7	99	0.505	0.918	0.453	0.213
Holotricha (% total protozoa)	1.86	2.71	1.07	1.24	1.17	1.86	1.26	1.77	2.28	1.04	0.505	0.918	0.453	0.213

1 *T*: *p* value of the type of tannin effect, CT vs. HT.

2 *L*: *p* value of the level of inclusion effect.

3 *T*×*L*: *p* value of the interaction of the fixed effects.

After sequencing, 130,841 ± 24,058 reads were obtained per sample, with a minimum of 101,733 reads and a maximum of 183,156. These values ensure that the results show complete representativeness of the microbiome. The mean number of OTUs detected (standardized value at 130,000 reads) was 8,800 ± 1,100 with a minimum of 7,500 and a maximum of 11,990. Shannon’s diversity index, whose value in the various experiments was between 8.26 and 9.48, was not significantly affected by the tannins.

**Figure 1:**
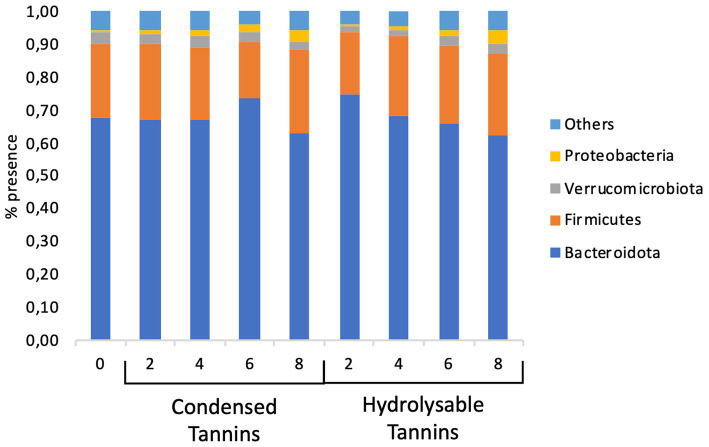
Percentage of the major phyla present in the bacterial population.

Globally, bacterial species belonging to 19 *phyla* have been identified (Supplementary Table 1). However, only 4 of these *phyla* (Bacteroidota, Firmicutes, Verrucomicrobiota, and Proteobacteria) were present in a percentage greater than 1% in at least all concentrations tested in a single treatment (CTs or HTs); finally, these 4 *phyla* always covered at least 94.1% of the bacterial population (Figure 1).

Archaea were present in the different samples to varying degrees between 0.21 and 1.21% of the total microbiota (data not shown); however, their presence was not affected by the treatments. Analysis of similarities (ANOSIM) detected no significant differences in bacterial diversity between treatments.

**Figure 2:**
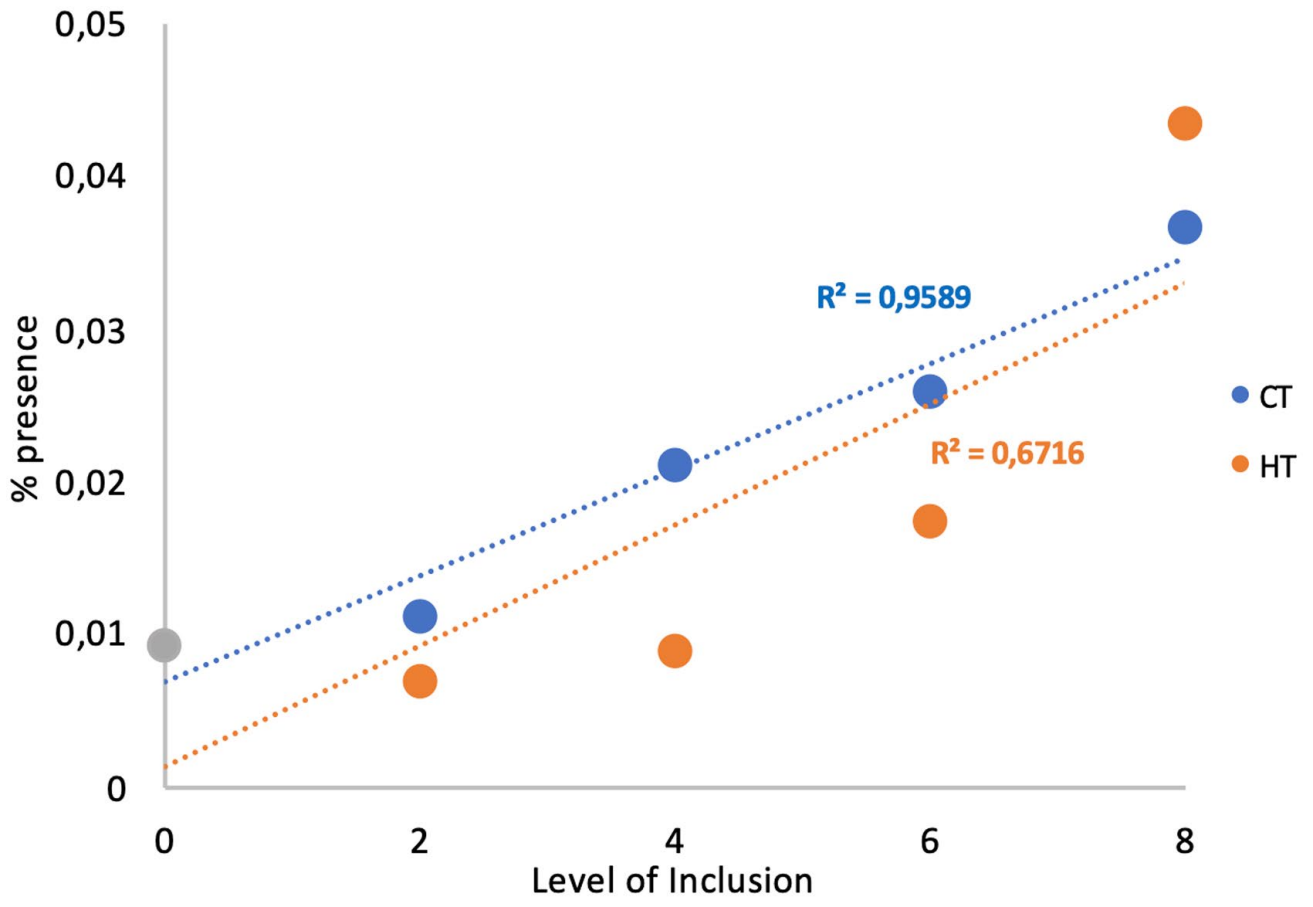
Relationship between the level of the treatments and proteobacteria.

The analysis of the relationship between the level of tannins and the abundance of the *phyla* highlighted a positive relationship for Proteobacteria (R^2^ = 0.959 and R^2^ = 0.672 for CTs and HTs, respectively; Figure 2).

The analysis of the microbiota at the class level showed the identification of bacteria belonging to 123 different classes. However, only 16 of these were present in a percentage higher than 0.5% in all treatments (Table 1). These bacterial classes covered a variable percentage between 73.2 and 85% of the entire microbial population.

**Figure 3:**
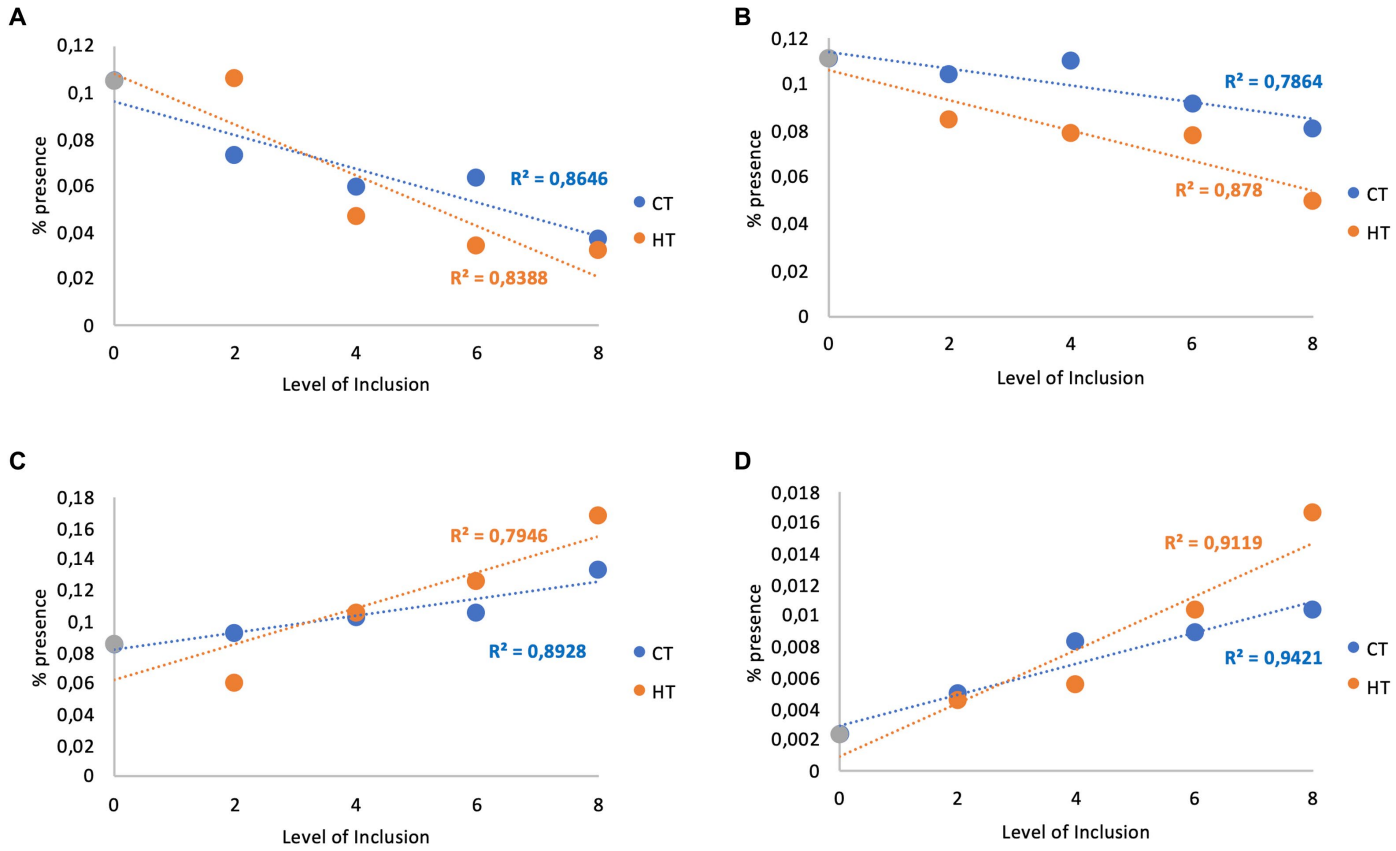
Correlation between tannins and families Bacteroidales_BS11 and F082 and genera Prevotella and Succinivibrio*. *: Correlation between the level of inclusion of condensed (CT) and hydrolysable (HT) tannin extracts and the percentage of the presence of families Bacteroidales_BS11 **(A)** and F082 **(B)** and genera Prevotella **(C)** and Succinivibrio **(D)**.

Regarding the correlation between the treatments and the percentage presence of the different microorganisms, some of them showed a close correlation between treatment and the percentage presence, and it is possible to highlight negative correlations for the families Bacteroidales_BS11 and F082 (Figures 3A,B) and positive correlations for the genera *Prevotella* and *Succinivibrio* (Figures 3C,D) valid for both types of tannins (CTs or HTs).

## 4. Discussion

In this *in vitro* experiment, which simulated ruminal fermentation, we tested both CTs and HTs purified extracts from quebracho and chestnut, respectively, at different levels of inclusion. Both types of tannins reduced the GP expressed as ml/g DM (0–48 h). The percentage reduction induced by both types of tannins at the different levels of inclusion was between 1.91 and 8.61% for CTs and between 1.44 and 5.74% for HTs. The reduction percentages of GP obtained in this study are similar to those observed by Jayanegara et al. (8) with tannins extracted from chestnut and quebracho at concentrations of 3.95, 5.92, and 7.89%, except for CTs at 8% for which level of inclusion we obtained an 8.61% reduction of GP while Jayanegara et al. (8) obtained a 13.4% reduction. Condensed tannins at 6% inclusion tended to reduce the GP by approximately 8.61% compared to the control, while HTs did not differentiate significantly from the control after Tukey’s adjustment.

Both types of tannins significantly reduced CH_4_ production at 24 h. Interestingly, during the second day of incubation (24–48 h), CTs continued to reduce CH_4_ production differently than HTs, with the result that CTs were more effective in reducing CH_4_ production than HTs during the 48 h of incubation. This can be due to the greater ruminal degradability of HTs compared to CTs (8), which leads to a reduction in the effect of HTs with the time of incubation. As reported by McSweeney et al. (19), HTs are more susceptible to microbial hydrolysis than condensed tannins, which agrees with the results of the present study. Overall, considering the regression equation for CTs (Table 3) and HTs (Table 4), the reduction in CH_4_ production (ml/g DM) during the 48 h of incubation was 17.5 and 10.4%, respectively, when tannins were added at 8% compared to the 0 level of inclusion. The same higher effect of CTs compared to HTs on CH_4_ production *in vitro* was observed by Bhatta et al. (17) but not by Hassanat and Benchaar (33), who achieved similar reductions in CH_4_ production when CTs from quebracho and HTs from chestnut were included at concentrations higher than 10% of the diet.

The reduction of rumen methanogenesis due to the presence of tannins can be caused by the following four mechanisms of action: direct effect on rumen methanogens, direct control of the protozoan population, reduction of diet digestibility, especially fiber degradability, and the sink action of hydrogen (18, 23, 34, 35). In our study, the reduction in CH_4_ production is probably due to the reduction in DMD more than the other proposed mechanisms; however, although at minor extent, the other proposed mechanisms of action cannot be excluded.

Indeed, in this experiment, archaeal and protozoan populations were not affected by the treatments; although, as report by Aboagye and Beauchemin (22), tannins can reduce their activity. Protozoa and methanogens have a symbiotic relationship in the rumen, and a high number of protozoa is often associated with higher CH_4_ production (36–38) since approximately 37% of total rumen methanogenesis is produced by protozoa-associated methanogens (39).

In the literature, it is possible to find different responses to the presence of tannins for both methanogens and protozoa, with variation due to the type and source of tannins; in addition, the substrates used could explain the different results reported despite the similar dose of tannins (17, 34, 40, 41). In this sense, concentrated rich substrates encourage the growth of protozoa (42, 43). Sarnataro and Spanghero (44) reported a depressive effect of chestnut tannins on protozoa when the substrate was ground corn meal but not when the substrate was a mixture of feeds, simulating a total mixed ration for ruminants. In our experiment, the significant effect of the type of tannin on total protozoa without an effect due to the level of inclusion is unclear.

Regarding the type of tannin, generally, it is stated that HTs has a greater effect on CH_4_ reduction compared to CTs due to the supposed different mechanism of action: HTs act directly on methanogens, inhibiting their growth or their activity, while CTs reduce CH_4_ more through the indirect effect caused by the reduction in fiber degradability (22, 45). In an *in vitro* study, Jayanegara et al. (8) compared two different HTs, chestnut and sumach tannins, with two CTs, mimosa and quebracho tannins, and contrary to our work, they observed a greater reduction in CH_4_ production with the addition of HTs. However, similar to our results, Jayanegara et al. (8) did not observe any significant differences in methanogen populations between the types of tannins, suggesting that a discrepancy may exist between CH_4_ production, the number of methanogens, their activity, and the number of protozoa. This discrepancy could also be explained by the reduction of the DMD, particularly of the fibrous fraction. In a recent paper, Foggi et al. (46) tested *in vitro* CTs from quebracho and HTs from chestnut (inclusion level of 2%), finding a CH_4_ reduction of approximately 10% for both CTs and HTs, without a significant reduction in the number of protozoa but accompanied by a reduction in digestibility.

Regarding the bacterial population, the increase in the genus *Prevotella* in the presence of tannins can be explained by its ability to tolerate tannins (47, 48) and also to a lower presence of competitors. Moreover, species of *Prevotella* are hydrogen-consuming bacteria that can produce propionate via the succinate pathway from the fermentation of sugar or via the acrylate pathway through the fermentation of lactate (49, 50). The reduction in CH_4_ production induced by tannins can lead to an increase in hydrogen concentration, which could promote the development of *Prevotella*. Additionally, species of *Prevotella* are noncellulolytic bacteria known to use ammonia for amino acid synthesis (51). Similar to what we have observed, Sarnataro and Spanghero (44) observed an increase in *P. ruminicola in vitro* associated with a decrease in rumen NH_3_ when chestnut HTs were added to the substrate. In line with these results, de Sant’ana et al. (52) reported an increase in the genus *Prevotella* when dairy goats were fed a diet including tannins. However, Carrasco et al. (14) observed a reduction effect of quebracho and chestnut tannins on *Prevotella* in an *in vivo* experiment with Holstein steers, although with a high degree of variance among animals.

We also observed an increase in the genus *Succinivibrio*, which belongs to the Succinivibrionaceae family and is responsible for the utilization of hydrogen to produce succinate, with tannin supplementation. Succinivibrionaceae and methanogens are mutually exclusive and could represent a potential target for a CH_4_ mitigation strategy (53). Contrary to what was reported in several works (8, 22, 45), the present study did not reveal a significant difference between tannin sources (CTs and HTs) when added at the same concentration in terms of negative effects on DMD and VFA production. Hassanat and Benchaar (33) reported a significant reduction (−6%) in VFA production with quebracho and chestnut tannins when added at concentrations higher than 5%. In our study, we recorded a linear reduction in total VFA production for both types of tannins. Several *in vitro* studies (17, 33, 54, 55) have reported a reduction in branched VFAs (isobutyrate, isovalerate and valerate) caused by CTs and HTs. Branched chain volatile fatty acids are a byproduct of amino acid deamination in the rumen, and in our experiment, the reduction in these branched VFAs was caused mainly by HTs. This observation is supported by the reduction in NH_3_ concentration that we registered for both CTs and HTs. According to the work of Hassanat and Benchaar (33), the NH_3_ concentration in the fermentation buffer at the end of the incubation was linearly reduced as the level of both quebracho and chestnut tannins increased, reaching a reduction of almost 17 and 18% with the inclusion level at 8% for CTs and HTs, respectively. Similar results were found by Foggi et al. (46), who observed a reduction in NH_3_ concentration of 13 and 10% for CTs and HTs, respectively, when included at 2%.

The reduction of GP, DMD and VFA, without changes in the relative quantities of acetate and propionate, could indicate, for both types of tannin, an effect of reducing the degradability more generally of the organic matter rather than the fiber. This is also to be seen in relation to changes in the microbiota, especially in the population of cellulolytic bacteria.

## 5. Conclusion

In this study, condensed tannins from quebracho were more effective in reducing CH_4_ production compared to HTs from chestnut. However, no significant changes in protozoan and archaeal populations were observed. Negative correlations with both CTs and HTs were observed with the families *Bacteroidales_BS11* and *F082*, while the correlations were positive for the genera *Prevotella* and *Succinivibrio*. Moreover, both types of tannins caused a reduction in GP, DMD and NH_3_ and linearly lowered VFA production as the level of tannins increased. This leads us to assume that the reduction in CH_4_ production is more related to the reduction in DMD caused by tannins than to their direct action on methanogens and protozoa. This could be detrimental to feed efficiency. On the other hand, the lower NH_3_ concentration observed with tannins may suggest a lower ruminal protein degradation, which would lead to a reduction of urinary nitrogen excretion and an increase in the faecal one, with favorable environmental and agronomic repercussions.

Due to the high variability of CTs and HTs, further studies investigating the chemical structure of the compounds and their mechanisms of action are needed to understand the different results observed in the literature.

Finally, *in vivo* studies will show possible long-term effects such as ruminal microbial adaptation.

## Data availability statement

The datasets presented in this study regarding the DNA sequences can be found in online repositories. The names of the repository/repositories and accession number(s) can be found at: https://doi.org/10.13130/RD_UNIMI/YYXP4V.

## Ethics statement

The animal study was reviewed and approved by University of Milan Ethics Committee for animal use and care and with the authorization of the Italian Ministry of Health, authorization no. 904/2016-PR.

## Author contributions

MB, SC, and LR: conceptualization. MB, SC, LR, PP, GG, and GMC: methodology and investigation. DP: cow surgery cannulation and animal care. MB and LR: validation and formal analysis. GMC, PP, and LR: resources. MB, SC, PP, GMC, and LR: data curation. MB and PP: writing – original draft preparation. MB, GC, SC, GG, MS, PP, SAZ, and LR: writing – review and editing. GMC and LR: supervision. LR and MM: project administration and funding acquisition. All authors contributed to the article and approved the submitted version.

## Funding

Project support was provided by the Regione Lombardia - PSR 2014–2020 Operazione 16.1.01 Gruppi Operativi PEI – d.d.s. 2951/2018.

## Conflict of interest

The authors declare that the research was conducted in the absence of any commercial or financial relationships that could be construed as a potential conflict of interest.

## Publisher’s note

All claims expressed in this article are solely those of the authors and do not necessarily represent those of their affiliated organizations, or those of the publisher, the editors and the reviewers. Any product that may be evaluated in this article, or claim that may be made by its manufacturer, is not guaranteed or endorsed by the publisher.
