# Supplementary material for: In vitro effects of different levels of quebracho and chestnut tannins on rumen methane production, fermentation parameters, and microbiota
Source: Front Vet Sci. 2023 Apr 18;10:1178288. doi: 10.3389/fvets.2023.1178288 (PMC10154982; doi:10.3389/fvets.2023.1178288)
Supplement: Supplementary file 1 [file Table_1.docx]

Supplementary Material

In vitro effects of different levels of quebracho and chestnut tannins on rumen gas and methane production, rumen fermentation parameters and microbiota

**Marco Battelli, Stefania Colombini, Pietro Parma, Gianluca Galassi, Gianni Matteo Crovetto, Mauro Spanghero, Davide Pravettoni, Sergio Aurelio Zanzani; Maria Teresa Manfredi, and Luca Rapetti^*^**

*** Correspondence:** Luca Rapetti: [luca.rapetti@unimi.it](mailto:luca.rapetti@unimi.it)

Supplementary Table 1: major *phyla* present in the bacterial population

|  | **Condensed Tannins (CT) extract** | | | | | **Hydrolysable Tannins (HT) extract** | | | | |
| --- | --- | --- | --- | --- | --- | --- | --- | --- | --- | --- |
| **% tannin extract:** | **0** | **2** | **4** | **6** | **8** | **0** | **2** | **4** | **6** | **8** |
| Bacteroidota | 0.675 | 0.668 | 0.672 | 0.733 | 0.631 | 0.675 | 0.745 | 0.680 | 0.661 | 0.620 |
| Firmicutes | 0.224 | 0.231 | 0.217 | 0.176 | 0.252 | 0.224 | 0.194 | 0.244 | 0.237 | 0.251 |
| Verrucomicrobiota | 0.037 | 0.030 | 0.033 | 0.025 | 0.023 | 0.037 | 0.018 | 0.020 | 0.029 | 0.027 |
| Bdellovibrionota | 0.009 | 0.012 | 0.012 | 0.010 | 0.009 | 0.009 | 0.005 | 0.009 | 0.008 | 0.008 |
| Synergistota | 0.006 | 0.006 | 0.006 | 0.003 | 0.005 | 0.006 | 0.003 | 0.004 | 0.005 | 0.004 |
| Proteobacteria | 0.009 | 0.011 | 0.021 | 0.026 | 0.037 | 0.009 | 0.007 | 0.009 | 0.017 | 0.044 |
| Spirochaetota | 0.009 | 0.010 | 0.011 | 0.009 | 0.016 | 0.009 | 0.007 | 0.008 | 0.011 | 0.013 |
| Elusimicrobiota | 0.003 | 0.004 | 0.005 | 0.003 | 0.002 | 0.003 | 0.003 | 0.004 | 0.003 | 0.003 |
| Desulfobacterota | 0.005 | 0.005 | 0.004 | 0.003 | 0.004 | 0.005 | 0.003 | 0.004 | 0.002 | 0.002 |
| Patescibacteria | 0.002 | 0.003 | 0.002 | 0.001 | 0.000 | 0.002 | 0.003 | 0.003 | 0.003 | 0.002 |
| Unknown Bacteria | 0.002 | 0.002 | 0.002 | 0.002 | 0.001 | 0.002 | 0.003 | 0.003 | 0.004 | 0.002 |
| Planctomycetota | 0.008 | 0.004 | 0.004 | 0.002 | 0.002 | 0.008 | 0.003 | 0.004 | 0.005 | 0.002 |
| Fibrobacterota | 0.002 | 0.006 | 0.004 | 0.003 | 0.006 | 0.002 | 0.002 | 0.003 | 0.007 | 0.006 |
| Euryarchaeota | 0.003 | 0.002 | 0.003 | 0.001 | 0.002 | 0.003 | 0.001 | 0.001 | 0.003 | 0.002 |
| Thermoplasmatota | 0.003 | 0.003 | 0.004 | 0.002 | 0.008 | 0.003 | 0.003 | 0.003 | 0.003 | 0.010 |
| Actinobacteriota | 0.000 | 0.000 | 0.000 | 0.000 | 0.000 | 0.000 | 0.000 | 0.001 | 0.000 | 0.000 |
| Armatimonadota | 0.000 | 0.000 | 0.000 | 0.000 | 0.000 | 0.000 | 0.000 | 0.000 | 0.000 | 0.000 |
| Chloroflexi | 0.001 | 0.001 | 0.000 | 0.000 | 0.000 | 0.001 | 0.000 | 0.000 | 0.001 | 0.000 |
| Cyanobacteia | 0.001 | 0.000 | 0.000 | 0.000 | 0.003 | 0.001 | 0.000 | 0.000 | 0.000 | 0.004 |
